# Comparison Between Single‐ and Multi‐slice Computed Tomography Body Composition Analysis in Patients With Oesophagogastric Cancer

**DOI:** 10.1002/jcsm.13673

**Published:** 2024-12-26

**Authors:** Leo R. Brown, Maria Soupashi, Michael S. Yule, Danielle R. Clyde, Ellen Gardner, Charlotte Smith, Ahmed Dhaif, Barry J. A. Laird, Stephen J. Wigmore, Richard J. E. Skipworth

**Affiliations:** ^1^ Clinical Surgery University of Edinburgh, Royal Infirmary of Edinburgh Edinburgh Scotland UK; ^2^ Institute of Genetics and Cancer University of Edinburgh, Western General Hospital Edinburgh Scotland UK; ^3^ St Columba's Hospice Edinburgh Scotland UK; ^4^ Department of General Surgery Victoria Hospital Kirkcaldy Scotland UK; ^5^ Department of General Surgery Forth Valley Royal Hospital Larbert Scotland UK; ^6^ Department of General Surgery Borders General Hospital Melrose Scotland UK; ^7^ Department of General Surgery Dumfries and Galloway Royal Infirmary Cargenbridge Scotland UK

**Keywords:** body composition, cachexia, gastric cancer, oesophageal cancer, survival

## Abstract

**Background:**

Single‐slice computed tomography (CT) body composition has been studied extensively for prognostication in patients with cancer. New software packages can also provide multi‐slice volumetric measurements, but the clinical utility of these remains under explored. This study aimed to evaluate the agreement between single‐ and multi‐slice body composition analyses in patients with oesophagogastric cancer and to explore the association between these measures and overall survival.

**Methods:**

Consecutive patients with newly diagnosed oesophagogastric (OG) cancer were identified through the prospectively maintained regional database of the South East Scotland Cancer Network across a 2‐year study period. CT body composition analyses were undertaken using scans collected during routine clinical care. Single‐slice (cross‐sectional area at mid L3) and multi‐slice (volume between T12 and L4) measurements were compared for skeletal muscle (SKM), subcutaneous adipose (SAT), visceral adipose (VAT) and intermuscular adipose (IMAT). Agreement between sex‐stratified z‐scores was quantified using Pearson correlation coefficients and Bland–Altman analyses. Cox proportional hazard modelling was used to estimate the effect of these measures on overall survival.

**Results:**

Overall, 504 patients (67.9% male, median 72 years) were newly diagnosed with OG cancer during the study period. Single‐ and multi‐slice (mean: 169 slices) measurements correlated highly for SKM (R: 0.97, *p* < 0.001), SAT (R: 0.98, *p* < 0.001), VAT (R: 0.97, *p* < 0.001), SKM radiodensity (R: 0.93, *p* < 0.001) and IMAT (R: 0.88, p < 0.001). Bias on Bland–Altman analysis was 0.00 for all tissue measurements. Limits of agreement (LoA) were narrowest for SAT (±0.43), VAT (±0.46) and SKM (±0.48), but slightly wider for SKM radiodensity (±0.73) and IMAT (±0.96). Adipose tissue ‘outliers’ (those where agreement between single‐ and multi‐slice z‐scores was outside the LoA) had a higher median weight and body mass index (BMI), suggestive of poorer agreement in patients with obesity. Sensitivity analysis, excluding those with BMI > 30, narrowed the LoA for SKM, VAT, SAT and IMAT. Direction and magnitudes of observed effect sizes for overall survival were all highly comparable, with hazard ratios for each tissue type varying by ≤ 0.04 between single‐ and multi‐slice adjusted estimates.

**Conclusions:**

Single‐slice and multi‐slice CT assessments provide highly correlated tissue measurements amongst patients with OG cancer. Associations between these measurements and overall survival were also comparable across both types of body composition analysis. Agreement between single‐ and multi‐slice measurements of adiposity is worse in patients with obesity, suggesting single‐slice analyses may less accurately reflect the quantity or distribution of adipose tissue in this patient group.

## 1 Introduction

Computed tomography (CT) scans are routinely performed as part of diagnosis, staging and surveillance in patients with cancer. This imaging provides vital information regarding the site and extent of malignant disease and has a pivotal role during multidisciplinary team (MDT) decision‐making. In addition to informing clinicians of tumour characteristics, CT can also be used to assess patients' body composition. Low cross‐sectional area [1] and radiodensity [2] of muscle are both known to be adversely prognostic across a multitude of cancer sites and stages. In patients with oesophagogastric (OG) malignancy, these measures have been consistently associated with reduced overall survival in patients with both resectable and un‐resectable OG malignancies [3, 4, 5, 6].

Previous studies have explored a variety of different methods for measuring muscle and adipose tissue via CT imaging. Amongst the most widely utilised is tissue cross‐sectional area, taken from a single axial slice at the middle of the third lumbar vertebra (L3) level. Body composition measurements at this level have previously been shown to correlate well with whole‐body estimates [7]. Early software programmes that facilitated such measurements were reliant on the user to manually segment tissues. This requires good training and anatomical knowledge for accurate estimates and can be labour intensive, limiting its application to larger‐scale studies. Commercially available alternatives have since been developed that can automate tissue segmentation across single‐slice axial images. The accuracy of these programmes has been validated across large cohorts [8], resulting in their widespread use in this research arena. Artificial intelligence has now facilitated further progress, with new software that is capable of multi‐slice (volumetric) body composition assessments across larger regions of interest. This technology has again performed well during validation testing against manual segmentation in an initial study of a small population of human patients (*n* = 50) [9], but its real‐world clinical utility remains under‐explored. To date, one study has assessed the concordance between single‐ and multi‐slice CT body composition measurements, and their association with survival, in a cohort of patients with operable colorectal cancer [10]. Multi‐slice CT body composition analysis is yet to be investigated in non‐curative populations or in patients with OG cancer.

This study aimed to evaluate the agreement between single‐slice (mid‐L3) and multi‐slice (T12–L4) body composition analyses in patients with newly diagnosed OG cancer, and to explore the association between these measures and overall survival. Our hypothesis was that the larger region of interest captured on multi‐slice (volumetric) assessment would provide a better estimate of whole‐body composition, compared with single‐slice analysis.

## 2 Methods

This cohort study retrospectively utilised patient‐level data and radiological imaging collected as part of routine clinical care. No changes were made to investigative or treatment pathways for the purposes of this work. Local institutional approval (Caldicott Guardian) was obtained prior to data collection. The manuscript is reported according to the STROBE guidelines [11].

### 2.1 Patient Cohort

Consecutive adult (≥ 18 years) patients were identified from the prospectively maintained, South East Scotland Cancer Network (SCAN) database. This database included those referred to the South East Scotland upper gastrointestinal cancer multidisciplinary team (MDT) with a new diagnosis of oesophageal, gastro‐oesophageal junction (GOJ) or gastric cancer, irrespective of planned treatment pathway (curative or non‐curative). A 2‐year study period was considered, between 1 January 2019 and 31 December 2020, which allowed all included patients to undergo a minimum of 3‐year follow‐up. Those referred to the MDT with a non‐malignant diagnosis or recurrence of a previously diagnosed OG cancer were not included. An additional known pre‐existing/concurrently diagnosed malignancy at an alternative site was a further exclusion criterion. Patients who did not undergo a computed tomography (CT) scan at the time of diagnosis or who had imaging that inadequately captured the region of interest were not eligible.

### 2.2 Clinical Staging Investigations

Patients routinely underwent an upper gastrointestinal endoscopy and thoraco‐abdominal CT scan during clinical staging, unless either investigation was contraindicated. Tumour stage was reported as per TNM classification (8th edition) and categorised according to American Joint Committee on Cancer (AJCC) clinical stage groupings [12, 13]. GOJ tumours were classified as per Siewert and Stein [14], based on findings at endoscopy. Type I and II GOJ tumours were categorised as oesophageal, and Type III GOJ tumours were considered to be gastric. Additional staging investigations (e.g., positron emission tomography [PET] CT scans or diagnostic laparoscopy and peritoneal washings‐based cytology) were performed selectively in line with national guidance.

### 2.3 Clinicopathological and Radiological Data Collection

Data were obtained from the SCAN database and via electronic patient records. This included basic demographics, American Society of Anaesthesiologists (ASA) grade [15], Charlson comorbidity index [16], Eastern Cooperative Oncology Group performance status (ECOG‐PS) [17], anthropometric measurements and disease characteristics. Patients were diagnosed as being obese if body mass index (BMI) ≥ 30 kg/m^2^ and underweight if BMI < 18.5 kg/m^2^ [18]. Involuntary weight loss, experienced prior to any treatment, and Malnutritional Universal Screen Tool (MUST) scores were recorded where available. Cachexia was defined using the Global Leadership Initiative on Malnutrition (GLIM) criteria for disease‐related malnutrition [19], in the presence of systemic inflammation [20]. Neutrophil‐to‐lymphocyte ratio (NLR), calculated by dividing the absolute neutrophil count by the absolute lymphocyte count, was selected as a marker of systemic inflammation. Although cut‐offs for ‘high’ NLR vary in the existing literature [21], a threshold of ≥ 3.5 was defined a priori [22]. A contrast‐enhanced, portal venous phase thoraco‐abdominal CT scan, obtained at clinical staging (prior to commencement of any treatment), was retrieved for all included patients. If this imaging was unavailable, or of insufficient quality for body composition analysis, patients were excluded from the cohort. Details of surgery, radiotherapy and systemic anti‐cancer therapies were obtained, alongside dates of death or last known follow‐up.

### 2.4 Body Composition Analyses

**FIGURE 1:**
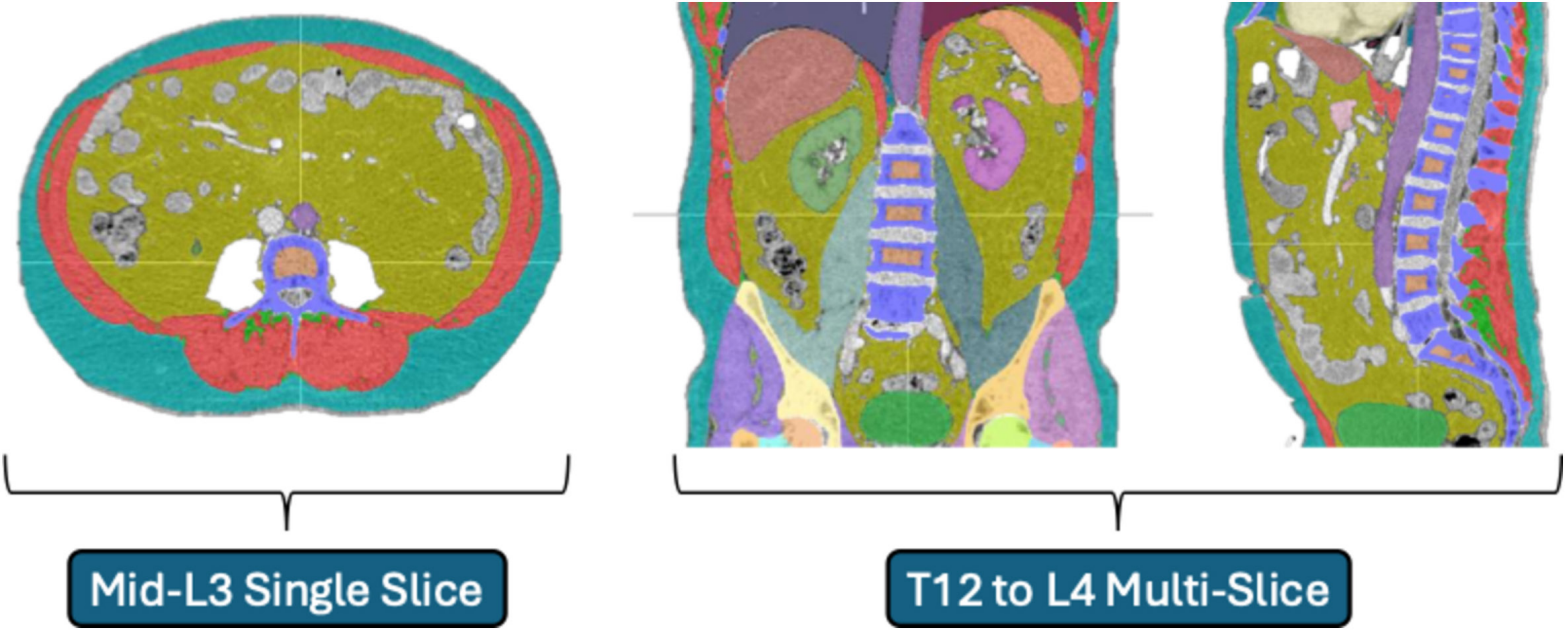
Comparison of body composition analysis at mid‐L3 and T12–L4 vertebral Levels. Note the segmented CT imaging displayed on coronal and sagittal planes (right) extends beyond T12–L4 region of interest analysed.

Body composition analyses were conducted using Data Analysis Facilitation Suite (DAFS Voronoi Health Analytics Inc., Vancouver, Canada, 2021). This software uses non‐linear image processing algorithms for automated segmentation of body tissues and annotation of axial vertebral levels. Although it has the function to assess numerous tissue types, those considered for the purposes of this study were skeletal muscle (SKM), subcutaneous adipose tissue (SAT), visceral adipose tissue (VAT) and intra‐muscular adipose tissue (IMAT). Segmentation and vertebral level annotation outputs were cross‐checked by a trained clinician investigator (L.R.B.) with manual edits completed as necessary. The presence of artefact or poor‐quality imaging was flagged at this time, with subsequent case exclusion as necessary. Multi‐slice measurements obtained were the respective volumes (cm^3^) of SKM, SAT, VAT and IMAT between the start of T12 and the end of L4 vertebral levels (Figure 1). This was selected as a region of interest that was consistently available on a single abdominal/pelvic CT series within this oesophagogastric patient population. It allowed comparison of tissue measurement data obtained across 5 entire vertebral levels (mean: 169 slices), against a single axial slice. Single‐slice measurements were cross‐sectional area (cm^2^) of the same tissue types at the mid‐L3 level. Mean radiodensity (HU) was also assessed for SKM across both multi‐slice and single‐slice analyses.

### 2.5 Statistical Analyses

Continuous data were summarised as mean and standard deviation (SD) or median and inter‐quartile range [IQR] based on visual and statistical evaluation for normality. Subsequent testing was performed via appropriate parametric or non‐parametric tests. Categorical data were cross‐tabulated, and differences were assessed with χ^2^ or Fisher's exact test. Sex‐stratified z‐scores (mean = 0 and SD = 1) were calculated for each tissue measurement on single‐ and multi‐slice analyses to allow unit‐free comparison. Pearson correlation coefficients were used to quantify the linear relationship between measurements for each tissue type. Bland–Altman plots were constructed to assess the bias (mean of differences) and upper/lower limits of agreement (LoA: bias ± 1.96 * SD). Patients were defined as ‘outliers’ where agreement between single‐ and multi‐slice z‐scores was outside of the upper or lower LoA from Bland–Altman plots for the respective tissue type. Sensitivity analyses were conducted: (1) patients with obesity (BMI ≥ 30.0) were excluded; (2) multi‐slice measurements were scaled to torso length (mm); and (3) tissue measurements from single‐slice and multi‐slice scans were normalised for height^2^ to create single‐ and multi‐slice skeletal muscle index (SMI: cross‐sectional area [cm^2^] or volume [cm^3^] /height^2^ [m^2^]). Multivariable regression, via Cox proportional hazards modelling, was used to estimate the association between single‐ or multi‐slice measurements and overall survival. Preliminary modelling was used to explore plausible confounders, and first‐order interactions were checked. Those selected for final model were based on minimisation of the Akaike information criterion (AIC). Multiple imputation by chained equations (MICE) was undertaken for missing data with analyses performed across 10 multiply imputed datasets and pooled using Rubin's rules [23]. Data analyses were undertaken using R 4.3.0 (R Foundation for Statistical Computing, Vienna, Austria) with *tidyverse, finalfit, blandr, eulerr, mice, finalpsm and survival* packages.

## 3 Results

Overall, 611 patients were considered from the primary dataset. Some were excluded owing to pre‐existing or concurrently diagnosed malignancy at an alternative site (*n* = 12) or recurrence of a previously diagnosed OG cancer (*n* = 40). A further group did not undergo a thoraco‐abdominal CT scan at the time of diagnosis (*n* = 12) or had issues with poor imaging quality/artefact that precluded accurate body composition analysis within the region of interest (*n* = 21). Of the remainder, several patients had CT scans that did not capture the entire T12‐L4 region of interest (*n* = 22). This left a final cohort of 504 patients, who were eligible for subsequent analyses.

### 3.1 Patient Characteristics and Treatment Pathways

The cohort contained 342 males (67.9%) and 162 females (32.1%) with a new diagnosis of OG cancer (Table 1). The median age across the cohort was 72 years (IQR: 64–80). A higher proportion of males were ASA grade 3 or 4 (54.1% vs. 45.1%, *p* = 0.028), but age, Charlson score and ECOG‐PS were all comparable between sexes. Median BMI, at the time of clinical staging, was greater amongst male patients (*p* = 0.002). No differences were identified between sexes regarding involuntary pre‐treatment weight loss or MUST score. Although tumour sites were comparable, there were a higher proportion of female patients with squamous cell carcinoma (32.7% vs. 12.9%, *p* < 0.001). Male sex was associated with more advanced disease on clinical staging (*p* = 0.007).

**TABLE 1:** Clinicopathological characteristics at diagnosis by sex.

		Male (*n* = 342)	Female (*n* = 162)	*p*
Age	Median [IQR]	71 [63–80]	73 [66–81]	0.099
ASA grade	1	17 (5.0)	18 (11.1)	0.028
	2	140 (40.9)	71 (43.8)	
	3	169 (49.4)	70 (43.2)	
	4	16 (4.7)	3 (1.9)	
Charlson comorbidity index	0–1	205 (59.9)	104 (64.2)	0.367
	2–4	114 (33.3)	52 (32.1)	
	≥ 5	23 (6.7)	6 (3.7)	
ECOG‐PS	0	115 (33.5)	57 (35.2)	0.965
	1	120 (35.1)	55 (34.0)	
	2	71 (20.8)	36 (22.2)	
	3	32 (9.4)	13 (8.0)	
	4	4 (1.2)	1 (0.6)	
Height (m)	Median [IQR]	1.7 [1.7–1.8]	1.6 [1.5–1.6]	< 0.001
	Missing data (%)	15 (4.4)	5 (3.1)	
Weight (kg)	Median [IQR]	80.2 [68.8–92.8]	62.2 [52.8–75.0]	< 0.001
	Missing data (%)	20 (5.8)	6 (3.7)	
BMI	Median [IQR]	26.9 [23.4–30.4]	24.7 [21.6–29.3]	0.002
	Missing data (%)	20 (5.8)	6 (3.7)	
Weight loss (kg)	Median [IQR]	4 [0–7]	4 [0–8]	0.429
	Missing data (%)	33 (9.6)	11 (6.8)	
NLR	Median [IQR]	3.7 [2.3–6.1]	3.3 [2.3–5.1]	0.094
	Missing data (%)	9 (2.6)	3 (1.9)	
Cachexia	Yes	153 (44.7)	68 (42.0)	0.626
	No	178 (52.1)	88 (54.3)	
	Missing data (%)	11 (3.2)	6 (3.7)	
MUST score	High risk	198 (57.9)	90 (55.6)	0.809
	Medium risk	48 (14.0)	23 (14.2)	
	Low risk	90 (26.3)	47 (29.0)	
	Missing data (%)	6 (1.8)	2 (1.2)	
Tumour site	Oesophagus	248 (72.5)	114 (70.4)	0.672
	Stomach	94 (27.5)	48 (29.6)	
Histology	Adenocarcinoma	273 (79.8)	97 (59.9)	< 0.001
	Squamous cell	44 (12.9)	53 (32.7)	
	None/negative	25 (7.3)	12 (6.8)	
Stage	I	1 (0.3)	2 (1.2)	0.007
	II	21 (6.1)	22 (13.6)	
	III	86 (25.1)	42 (25.9)	
	IV	216 (63.2)	83 (51.2)	
	Missing data (%)	18 (5.3)	13 (8.0)	

*Note:* Data displayed as number (%) unless stated otherwise. Clinical Staging as per American Joint Committee on Cancer (AJCC) groupings.

Abbreviations: ASA, American Society of Anaesthesiologists; BMI, body mass index; ECOG‐PS, Eastern Cooperative Oncology Group performance status; GLIM, Global Leadership Initiative in Malnutrition; GP, general practitioner; MUST, Malnutrition Universal Screening Tool; NLR, neutrophil‐to‐lymphocyte ratio.

**FIGURE 2:**
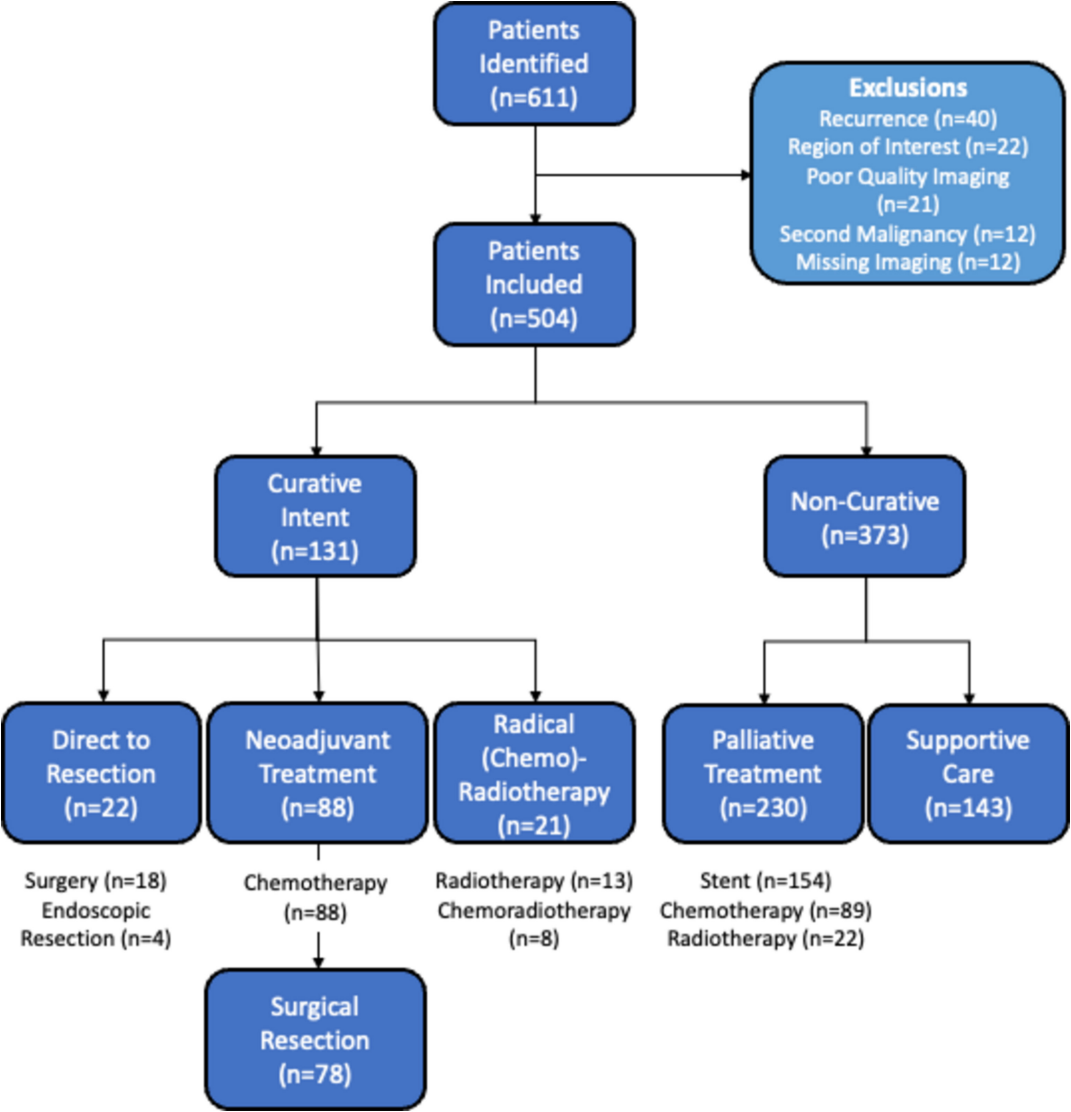
Flow chart demonstrating treatment pathways for the study cohort.

Treatment was commenced with curative intent for 131 patients (26.0%), but 373 patients (74.0%) were deemed unsuitable for curative treatment. Treatment pathways are depicted in Figure 2. Most patients treated with curative intent had neoadjuvant chemotherapy (NAC) with a plan for subsequent surgical resection (*n* = 88). Disease progression during NAC, identified radiologically (*n* = 6) or intra‐operatively (*n* = 4), precluded the planned OG cancer resection for 11.4% of these patients.

### 3.2 Comparison of Single‐ and Multi‐slice Methods of Body Composition Analysis

Males had significantly higher measures of SKM than females (mean volume: 2064 cm^3^ vs. 1308 cm^3^, mean area: 136 cm^2^ vs. 93 cm^2^, both *p* < 0.001; Appendix S1a). SMD was comparable between sexes on both single‐ and multi‐slice assessments. The volume and L3 cross‐sectional area of VAT were significantly higher in males (both *p* < 0.001). Females had more SAT, but this difference was only statistically significant on single‐slice measurements (mean volume: 2544 cm^3^ vs. 2407 cm^3^, *p* = 0.341, mean area: 189 cm^2^ vs. 168 cm^2^, *p* = 0.041). Conversely, increased IMAT in male patients was evident on multi‐ (*p* < 0.001) but not single‐slice assessments (*p* = 0.205). Comparison of cachectic and non‐cachectic patients revealed significantly decreased SKM, SAT and VAT on both multi‐slice and single‐slice measurements (Appendix S1b). Lower SMD was evident in patients with cachexia based on single (*p* = 0.003) but not multi‐slice measurements (*p* = 0.078).

**FIGURE 3:**
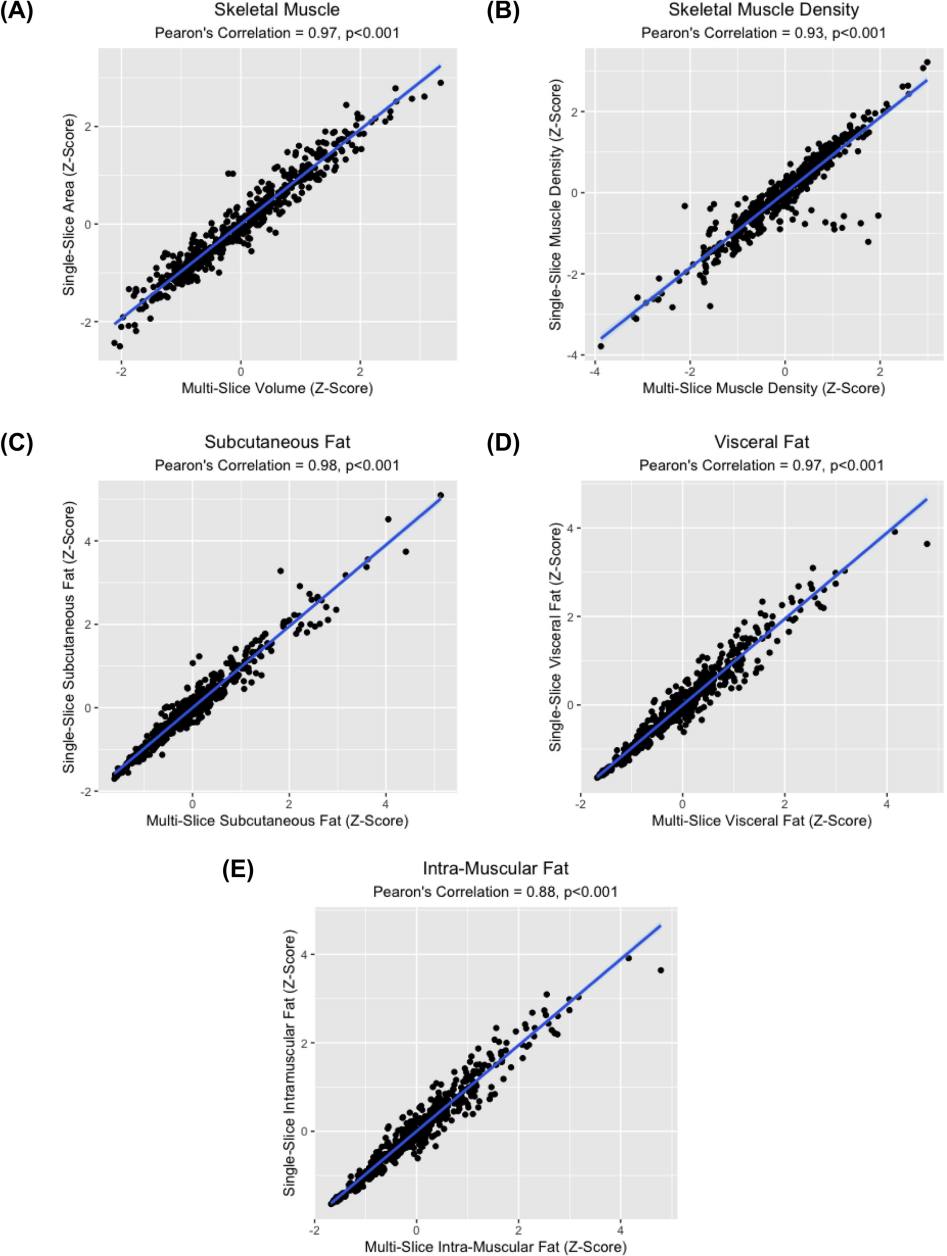
Scatter plots showing correlation between Z‐scores of single‐ and multi‐slice body composition estimates.

Positive correlations between sex‐stratified z‐scores for single‐slice and multi‐slice estimates were evident for all considered tissue measurements (Figure 3). These correlations were strongest for SKM (R: 0.97, *p* < 0.001), SAT (R: 0.98, *p* < 0.001) and VAT (R: 0.97, *p* < 0.001). High concordance was also seen for single‐ vs. multi‐slice comparison of SKM radiodensity (R: 0.93, *p* < 0.001); however, more outliers were visually evident. There was strong, yet lesser, correlation on comparison of IMAT measurements (R: 0.88, *p* < 0.001).

**FIGURE 4:**
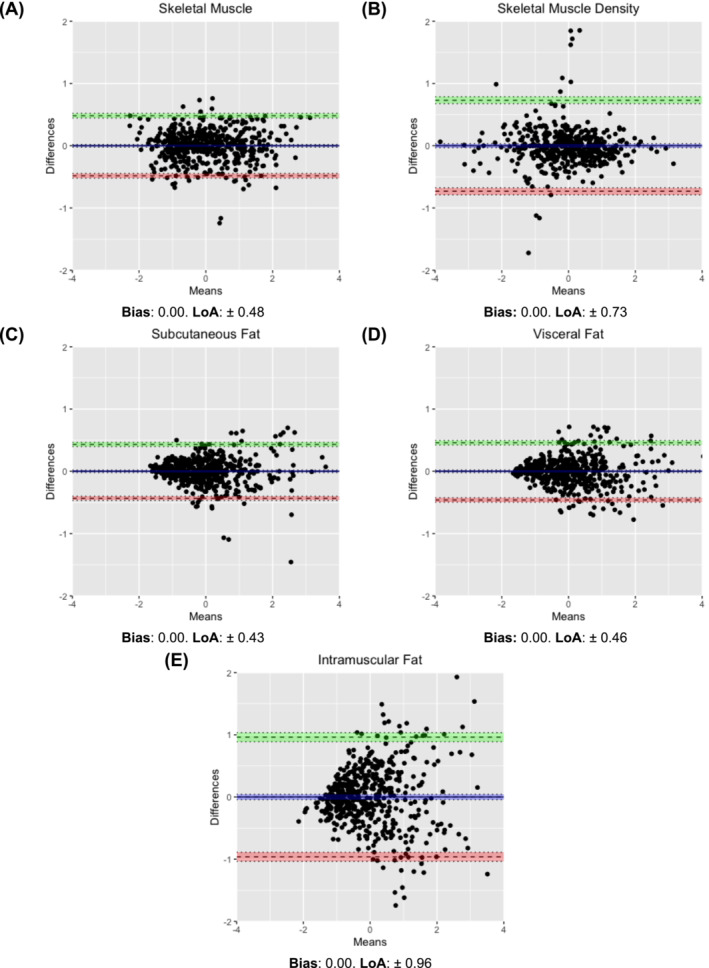
Bland–Altman plots showing agreement between Z‐scores of single‐ and multi‐slice body composition estimates with limits of agreement (LoA).

Agreement was further analysed using Bland–Altman plots (Figure 4). The bias (mean difference) between the z‐scores of single‐ and multi‐slice assessments was 0.00 for all tissue measurements. LoA were narrowest for SKM (±0.48, 95% CI: ±0.45–0.52), SAT (±0.43, 95% CI: ±0.40–0.47) and VAT (±0.46, 95% CI: ±0.42–0.49) but slightly wider for SKM radiodensity (±0.73, 95% CI: ±0.68–0.79) and IMAT (±0.96, 95% CI: ±0.89–1.04).

### 3.3 Outlier Patients Characteristics

**FIGURE 5:**
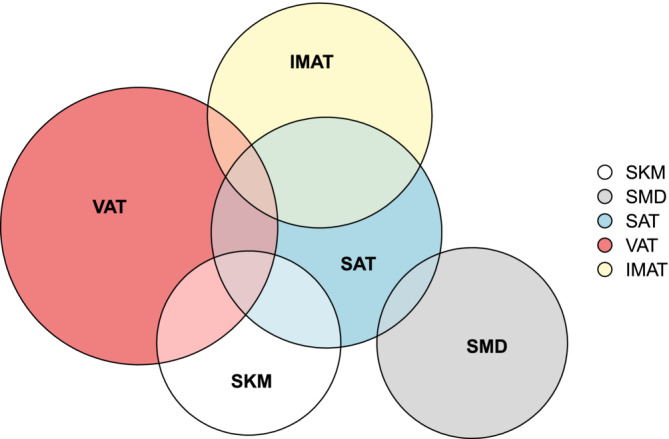
Euler diagram of patients noted as outliers for any body composition tissue measurement (*n* = 90).

Visual inspection of the Bland–Altman plots suggested more widely scattered difference values as the mean increased (heteroscedasticity), particularly amongst adipose tissue measurements. To investigate this further, the characteristics of ‘outliers’ (those outside the limits of agreement for a particular tissue measurement) were compared to the remainder. Ninety patients (17.9%) had at least one outlier tissue measurement. Of these, 30.0% (*n* = 27) were outliers for more than one tissue type, with particular overlap between subcutaneous fat and other tissue types (Figure 5).

Clinicopathological features amongst outliers for SKM were comparable with the rest of the cohort (Appendix S2a). Median NLR was higher amongst SMD outliers (5.4 vs. 3.5, *p* = 0.015) and a greater proportion were cachectic, as per the GLIM diagnostic criteria with evidence systemic inflammation (80% vs. 44.3%, *p* = 0.008).

On examination of adipose measurement outliers, a number of differences were evident (Appendix S2b). A greater proportion of SAT outliers were female (52.2% vs. 31.2%, *p* = 0.041). Median weight (89.9 kg vs. 74.6 kg, *p* = 0.005) and BMI (34.4 kg/m^2^ vs. 25.9 kg/m^2^, *p* < 0.001) were also higher amongst SAT outliers (Appendix S3). Increased weight and BMI were also observed amongst VAT and IMAT outliers (all *p* < 0.001). This pattern is suggestive of poorer agreement between single‐ and multi‐slice measurements of adiposity in patients with obesity.

### 3.4 Sensitivity Analysis: Exclusion of Patients With Obesity

Analyses were repeated for the ‘non‐obese’ subgroup of patients with BMI < 30.0 (*n* = 356, Table 2). Narrowed limits of agreement were evident on Bland–Altman analysis for SKM (bias: 0.00, LoA: ±0.46), SAT (bias: −0.02, upper LoA: 0.34, lower LoA: −0.38), VAT (bias: 0.00, upper LoA: 0.40, lower LoA: −0.41) and IMAT (bias: −0.10, upper LoA: 0.78, lower LoA −0.97). Agreement for SMD (bias: 0.04, upper LoA: 0.83, lower LoA: −0.74) was not improved. Further subgroup analyses were performed with patients stratified by BMI groups (Appendix S4 *)*.

**TABLE 2:** Comparison of agreement between Z‐scores of single‐ and multi‐slice body composition estimate for all patients (*n* = 504) vs. non‐obese cohort (*n* = 356).

	Pearson correlation	Bland–Altman analysis		
		*Bias*	*Upper LoA*	*Lower LoA*
Skeletal muscle *(All patients)*	0.97 (*p* < 0.001)	0.00 (−0.02 to 0.02)	0.48 (0.45 to 0.52)	−0.48 (−0.52 to −0.45)
*(Non‐obese cohort)*	0.96 (*p* < 0.001)	0.00 (−0.02 to 0.02)	0.46 (0.42 to 0.50)	−0.46 (−0.50 to −0.42)
Radiodensity *(All patients)*	0.93 (*p* < 0.001)	0.00 (−0.03 to 0.03)	0.73 (0.68 to 0.79)	−0.73 (−0.79 to −0.68)
*(Non‐obese cohort)*	0.91 (*p* < 0.001)	0.04 (0.00 to 0.09)	0.83 (0.76 to 0.90)	−0.74 (−0.82 to −0.67)
Subcutaneous fat *(All patients)*	0.98 (*p* < 0.001)	0.00 (−0.02 to 0.02)	0.43 (0.40 to 0.47)	−0.43 (−0.47 to −0.40)
*(Non‐obese cohort)*	0.97 (*p* < 0.001)	−0.02 (−0.04 to 0.00)	0.34 (0.31 to 0.37)	−0.38 (−0.41 to −0.34)
Visceral fat *(All patients)*	0.97 (*p* < 0.001)	0.00 (−0.02 to 0.02)	0.46 (0.42 to 0.49)	−0.46 (−0.49 to 0.42)
*(Non‐obese cohort)*	0.97 (*p* < 0.001)	0.00 (−0.02 to 0.02)	0.40 (0.37 to 0.45)	−0.41 (−0.45 to −0.37)
Intramuscular fat *(All patients)*	0.86 (*p* < 0.001)	0.00 (−0.05 to 0.05)	0.96 (0.89 to 1.04)	−0.96 (−1.04 to −0.89)
*(Non‐obese cohort)*	0.88 (*p* < 0.001)	−0.10 (−0.14 to −0.05)	0.78 (0.70 to 0.86)	−0.97 (−1.05 to −0.89)

*Note:* Bland–Altman analyses presented with 95% confidence intervals in parenthesis.

Additional sensitivity analyses were conducted with multi‐slice tissue measurements scaled by torso length and normalising measurements for height squared (Appendix S5). The results of these were highly comparable to those described above, with no clear improvement in agreement between single‐ and multi‐slice measurements following either adjustment.

### 3.5 Comparison of Single‐Slice vs. Multi‐Slice Body Composition's Association With Overall Survival

The median follow‐up period across the cohort was 47 months (range: 36–59). Median survival was over 3 ½ years (1295 days) in those treated curatively, approximately 7 months (216 days) in those treated palliatively and 63 days amongst patients who received best supportive care only.

Single‐ and multi‐slice body composition measurements were analysed in isolation for their association with overall survival, following imputation and adjustment for confounders (age, sex, ASA grade, tumour site and stage). Direction and magnitudes of observed effect sizes were all highly comparable, with hazard ratios for each tissue type varying by ≤ 0.04 between single‐ and multi‐slice adjusted estimates (Table 3). Both increased cross‐sectional area (aHR 0.57 [95% CI: 0.49–0.67], *p* < 0.001) and volume (aHR: 0.61 [95% CI: 0.52–0.71], *p* < 0.001) of SKM were associated with improved overall survival. Significant associations were also detected between longer survival and greater area/volume of SAT and VAT. When analyses were stratified by treatment intent subgroups, similarly comparable effect sizes were observed across single‐ and multi‐slice estimates for all tissue types (Appendix S6).

**TABLE 3:** Comparison of association between single‐ and multi‐slice body composition and overall survival.

	L3 single‐slice (cross‐sectional area)				Multi‐slice (volume)			
	Univariable HR (95% CI)	*p*	Multivariable HR (95% CI)	*p*	Univariable HR (95% CI)	*p*	Multivariable HR (95% CI)	*p*
SKM Area/volume	0.75 (0.68–0.83)	< 0.001	0.57 (0.49–0.67)	< 0.001	0.78 (0.70–0.85)	<0.001	0.61 (0.52–0.71)	< 0.001
SKM Radiodensity	0.86 (0.78–0.94)	0.002	1.00 (0.89–1.11)	0.930	0.88 (0.80–0.97)	0.008	1.04 (0.93–1.16)	0.506
SAT Area/volume	0.83 (0.74–0.92)	0.001	0.82 (0.73–0.92)	< 0.001	0.84 (0.76–0.94)	0.002	0.83 (0.74–0.94)	0.003
VAT Area/volume	0.85 (0.77–0.94)	0.001	0.75 (0.67–0.85)	< 0.001	0.84 (0.76–0.92)	< 0.001	0.73 (0.65–0.83)	< 0.001
IMAT Area/volume	1.01 (0.92–1.11)	0.760	0.91 (0.82–1.01)	0.071	0.99 (0.90–1.09)	0.814	0.89 (0.79–0.99)	0.040

*Note:* Each tissue measurement has modelled in isolation with adjustment for confounders but not for other tissue measurements.

Abbreviations: CI, confidence interval; HR, hazard ratio; IMAT, intramuscular adipose tissue; L3, third lumbar vertebra; SAT, subcutaneous adipose tissue; SKM, skeletal muscle; VAT, visceral adipose tissue.

### 3.6 Subgroup Analysis by Obesity Status

In light of the findings from the BMI‐based sensitivity analysis, two further regression models were constructed for patients without and with obesity (BMI ≥ 30.0). In the non‐obese subgroup (Appendix S7a), the protective effect of increased SKM area (aHR: 0.58 [95% CI: 0.48–0.71], *p* < 0.001) and volume (aHR: 0.61 [95% CI: 0.50–0.76], *p* < 0.001) remained almost identical to that overall cohort. In the obese subgroup (Appendix S7b), that association was significant but slightly weaker. Although increased SAT area (aHR: 0.68 [95% CI: 0.57–0.81], *p* < 0.001) and volume (aHR: 0.70 [95% CI: 0.58–0.85], *p* < 0.001) were protective in patients without obesity, an opposing effect was evident in the high BMI group for both area (aHR: 1.24 [95% CI: 1.01–1.52], *p* = 0.043) and volume (aHR 1.29 [95% CI: 1.05–1.57], *p* = 0.015). The protective effect of high VAT and IMAT was also only evident in the non‐obese subgroup.

## 4 Discussion

This study has confirmed that single‐slice (mid L3) and multi‐slice (T12 to L4) assessments of CT body composition provide highly correlated measurements for skeletal muscle, subcutaneous fat, visceral fat and intra‐muscular fat amongst patients with OG cancer. Furthermore, the associations between these tissues measurements and overall survival are comparable across both types of body composition analysis. On closer inspection of those patients where agreement between single‐ and multi‐slice measurements was less strong, a greater proportion were noted to be obese. As such, it is likely that single‐slice measurements are less able to accurately reflect the quantity or distribution of adipose tissue in some patients with excess fat accumulation.

The influence of CT body composition on clinical outcomes for patients with cancer has been extensively studied. Much of this interest stems from attempts to identify cachexia; a syndrome characterised by the loss of muscle, with or without fat [24]. Cachexia is known to have devastating effects on both the quality and quantity of remaining life and is highly prevalent in patients with oesophageal and gastric cancer [25], including even those with curable disease [26]. Although traditionally this syndrome was simply characterised by involuntary weight loss, interrogation of radiological imaging allows us to now quantify the tissue types that have contributed towards that loss. It is known that many patients with a higher body weight, in whom weight loss can be more occult, will concurrently have low radiological measurements of muscularity. This combination, termed ‘sarcopenic obesity’, would likely remain unidentified with simple anthropometric assessments, yet has a particularly adverse influence on prognosis [27]. Differing patterns of muscle and fat wasting have also been described on serial imaging, suggestive of multiple distinct cachectic phenotypes. The improved granularity achievable through CT body composition analysis is therefore of great value to clinicians and researchers [28].

Anyene et al. recently investigated the concordance of multi‐slice CT body composition in patients with colorectal cancer in the United States [10]. Their patient cohort were all < 80 years of age and had non‐metastatic disease amenable to surgical resection with curative intent. As such, they represented a more select group of patients with cancer, amongst whom the prevalence of cachexia was likely to be lower than that of the present study's mixed OG cohort [29]. On comparison of results from the two studies, Bland–Altman measures of concordance between single‐ and multi‐slice body composition estimates were almost identical for SAT and VAT. Skeletal muscle area/volume had a narrower limit of agreement in the OG cohort than the colorectal cohort (±0.48 vs. ±0.78). In both studies, IMAT measurements were the least concordant (OG LoA: ±0.96 and colorectal LoA: ±0.92). Although Anyene et al. evaluated T12‐L5 multi‐slice measurements on abdominal CT, preliminary inspection of this cohort's scans found that the entirety of the L5 vertebral level was frequently not captured during their routine clinical imaging. This variation may represent a difference in practice between the United States and the United Kingdom or may simply be reflective of the primary cancer in question being sited superiorly within the abdomen/thorax. It should also be noted, however, that inclusion of the L5 vertebra could present difficulties with identifying where the region of interest ends in patients affected by sacralisation of the L5 vertebra (estimated prevalence ~ 1 in 10) [30]. As such, we would suggest that the comparable findings of the present study could justify T12–L4 as a pragmatic and appropriate region of interest for future investigation of multi‐slice body composition.

Results from this OG cancer cohort highlighted a significant association between low quantity of skeletal muscle and poorer overall survival. This is in keeping with the existing literature where decreased skeletal muscle area/index has been consistently linked to worse patient outcomes [3, 4, 5, 6]. Decreased measurements of skeletal muscle radiodensity are also thought to be adversely prognostic [2]; however, this effect was not evident amongst our patient cohort following adjustment for confounding variables. SMD ‘outliers’ in the present study were noted to have a higher median NLR, and a greater proportion met the GLIM criteria for cachexia, which requires evidence of systemic inflammation. This is suggestive of less agreement between single‐ and multi‐slice measurements in patients who are more inflamed. The considerable influence that systemic inflammation can have on body composition analysis has previously been demonstrated by our group [31] with resultant oedema thought to influence estimates of tissue radiodensity. As such, it could be suggested that consideration of a larger region of interest, via multi‐slice CT body composition, may be beneficial for more accurate estimation of tissue radiodensity amongst patients with evidence of systemic inflammation.

The effect of CT‐measured adiposity on survival in patients with cancer is less clear [32]; however, it has been reported that survival in patients with fat wasting alone can be as short as those with both muscle and fat wasting [28]. Debate exists as to whether degrees of obesity can even confer a survival advantage in some patients with cancer [33]. In this cohort, a protective effect of increased SAT measurements was evident in non‐obese patients, where in the obese subgroup, more SAT was detrimental. This may represent a non‐linear relationship where either insufficient or excessive adiposity can be poor prognostic markers.

‘Outliers’ identified as having poorly concordant single‐ vs. multi‐slice SAT /VAT/IMAT estimates were more commonly patients with obesity. Therefore, it could be postulated that measurements from single‐slice CT body composition analysis are not able to accurately characterise adiposity in patients with obesity. This is a particularly important consideration for patients with oesophageal or gastric adenocarcinoma, as known obesity‐related cancer sites [34]. Measures of adiposity are clearly of consequence in patients with cancer, and we would suggest that their accurate estimation is as important as SKM measurements.

Low cross‐sectional area or volume of tissue on body composition analysis, when identified at a single time point, may either be reflective of constitutional low muscularity (age‐related, insidious sarcopenic changes) or new dynamic wasting (disease‐related malnutrition or cancer cachexia). Both of these may be expected to be adversely prognostic, but the pathophysiology and effect size associated will likely differ. Although multi‐slice analysis may represent a step forward in terms of improving the ability of that ‘snapshot’ to demonstrate a patient's body habitus, one scan cannot inform us of a patient's trajectory. Body composition should be considered longitudinally or in combination with other important markers of the cachectic phenotype (e.g., weight loss, biomarkers, functional and dietary status) to better appreciate the host's disease response. Future studies should consider in more detail how longitudinally changes in multi‐slice body composition, for example, following NAC, may be associated with short‐ and long‐term patient outcomes. The retrospective nature of the present study is also a limitation, and this precludes the availability of non‐routinely collected clinical data. Future research pertaining to body composition in other cancer sites and geographical regions would be valuable, where the influence of potentially influential factors such as ethnicity may be more readily explored. This manuscript focussed on muscle and fat captured via abdominal CT scans, as the more commonly studied measurements in patients with gastrointestinal cancer. Other tissue types and alternative regions of interest (e.g., thoracic) should be considered for exploration in future studies.

## 5 Conclusion

Single‐ and multi‐slice measurements are largely comparable for estimating body composition amongst patients with OG cancer, irrespective of disease stage. Multi‐slice analyses appear to be advantageous for accurate estimation of adiposity in patients with obesity. Future research should aim to further investigate the value of CT body composition analysis across other anatomical regions of interest and tissue types. Although the measurements considered in the present study appear to be associated with important clinical outcomes, changes in other body composition features may have even greater clinical utility.

## Conflicts of Interest

Leo R. Brown, Maria Soupashi, Michael S. Yule, Danielle R. Clyde, Ellen Gardner, Charlotte Smith, Ahmed Dhaif and Stephen J. Wigmore declare no conflicts of interest. Richard J.E. Skipworth has received personal fees for consultancy from Avidity Biosciences, Actimed, Faraday and Helsinn. Barry J.A. Laird has received personal fees for consultancy from Artelo, Actimed, Faraday, Kyona Kirin and Toray.

## Supporting information


**Appendix S1:** Body Composition Analysis.
**Appendix S2:** Comparison of Clinicopathological Characteristics = Between Outliers and Non‐Outliers for Each Respective Tissue Type.
**Appendix S3:** Bland–Altman Plots Showing Agreement Between Z‐Scores of Single‐ & Multi‐Slice Body Composition Estimates with Limits of Agreement (LoA) with BMI Colour Scale.
**Appendix S4:** Comparison of Agreement Between Z‐Scores of Single‐ and Multi‐Slice Body Composition Estimates with Patients Stratified by BMI.
**Appendix S5:** Comparison of Agreement Between Z‐Scores of Single‐ and Multi‐Slice Body Composition Estimate (Whole Cohort vs. Scaled Estimates).
**Appendix S6:** Comparison of Association Between Single‐ and Multi‐Slice Body Composition and Overall Survival.
**Appendix S7:** Supporting Information.

## Data Availability

Anonymised data may be available from the corresponding author upon reasonable request.

## References

[1] S. S. Shachar , G. R. Williams , H. B. Muss , and T. F. Nishijima , “Prognostic Value of Sarcopenia in Adults With Solid Tumours: A Meta‐Analysis and Systematic Review,” European Journal of Cancer 57 (2016): 58–67. 26882087 10.1016/j.ejca.2015.12.030

[2] G. F. P. Aleixo , S. S. Shachar , K. A. Nyrop , H. B. Muss , L. Malpica , and G. R. Williams , “Myosteatosis and Prognosis in Cancer: Systematic Review and Meta‐Analysis,” Critical Reviews in Oncology/Hematology 145 (2020): 102839. 31877534 10.1016/j.critrevonc.2019.102839

[3] U. M. Jogiat , H. Sasewich , S. R. Turner , et al., “Sarcopenia Determined by Skeletal Muscle Index Predicts Overall Survival, Disease‐Free Survival, and Postoperative Complications in Resectable Esophageal Cancer: A Systematic Review and Meta‐Analysis,” Annals of Surgery 276 (2022): e311–e318. 35794004 10.1097/SLA.0000000000005452

[4] U. M. Jogiat , E. L. R. Bédard , H. Sasewich , et al., “Sarcopenia Reduces Overall Survival in Unresectable Oesophageal Cancer: A Systematic Review and Meta‐Analysis,” Journal of Cachexia, Sarcopenia and Muscle 13, no. 6 (2022): 2630–2636, 10.1002/jcsm.13082. 36151845 PMC9745498

[5] S. K. Kamarajah , J. Bundred , and B. H. L. Tan , “Body Composition Assessment and Sarcopenia in Patients With Gastric Cancer: A Systematic Review and Meta‐Analysis,” Gastric Cancer 22 (2019): 10–22. 30276574 10.1007/s10120-018-0882-2

[6] P. Fang , J. Zhou , X. Xiao , et al., “The Prognostic Value of Sarcopenia in Oesophageal Cancer: A Systematic Review and Meta‐Analysis,” Journal of Cachexia, Sarcopenia and Muscle 14 (2023): 3–16. 36415154 10.1002/jcsm.13126 PMC9891912

[7] M. Mourtzakis , C. M. M. Prado , J. R. Lieffers , T. Reiman , L. J. McCargar , and V. E. Baracos , “A Practical and Precise Approach to Quantification of Body Composition in Cancer Patients Using Computed Tomography Images Acquired During Routine Care,” Applied Physiology, Nutrition, and Metabolism 33 (2008): 997–1006. 10.1139/H08-075 18923576

[8] E. M. Cespedes Feliciano , K. Popuri , D. Cobzas , et al., “Evaluation of Automated Computed Tomography Segmentation to Assess Body Composition and Mortality Associations in Cancer Patients,” Journal of Cachexia, Sarcopenia and Muscle 11 (2020): 1258–1269. 32314543 10.1002/jcsm.12573 PMC7567141

[9] D. Ma , V. Chow , K. Popuri , and M. F. Beg , “Comprehensive Validation of Automated Whole Body Skeletal Muscle, Adipose Tissue, and Bone Segmentation from 3D CT images for Body Composition Analysis: Towards Extended Body Composition [Internet],” arXiv, (2021), accessed October 10, 2022, http://arxiv.org/abs/2106.00652.

[10] I. Anyene , B. Caan , G. R. Williams , et al., “Body Composition From Single Versus Multi‐slice Abdominal Computed Tomography: Concordance and Associations With Colorectal Cancer Survival,” Journal of Cachexia, Sarcopenia and Muscle 13 (2022): 2974–2984. 36052755 10.1002/jcsm.13080 PMC9745558

[11] E. von Elm , D. G. Altman , M. Egger , S. J. Pocock , P. C. Gøtzsche , and J. P. Vandenbroucke , “Strengthening the Reporting of Observational Studies in Epidemiology (STROBE) Statement: Guidelines for Reporting Observational Studies,” BMJ 20, no. 335 (2007): 806–808. 10.1136/bmj.39335.541782.AD PMC2034723 17947786

[12] T. W. Rice , D. T. Patil , and E. H. Blackstone , “8th Edition AJCC/UICC Staging of Cancers of the Esophagus and Esophagogastric Junction: Application to Clinical Practice,” Annals of Cardiothoracic Surgery 6 (2017): 119–130. 28447000 10.21037/acs.2017.03.14 PMC5387145

[13] M. Amin , F. Greene , S. Edge , C. Compton , J. Gershenwald , and R. Brookland , “The Eighth Edition AJCC Cancer Staging Manual: Continuing to Build a Bridge From a Population‐Based to a More ‘Personalized’ Approach to Cancer Staging,” CA: A Cancer Journal for Clinicians 67 (2017): 93–99. 28094848 10.3322/caac.21388

[14] J. R. Siewert and H. J. Stein , “Carcinoma of the Gastroesophageal Junction ‐ Classification, Pathology and Extent of Resection,” Diseases of the Esophagus 9 (1996): 173–182.

[15] R. D. Dripps , “New Classification of Physical Status,” Anesthesiology 24 (1963): 111.

[16] M. E. Charlson , P. Pompei , K. L. Ales , and C. R. MacKenzie , “A New Method of Classifying Prognostic Comorbidity in Longitudinal Studies: Development and Validation,” Journal of Chronic Diseases 40 (1987): 373–383. 3558716 10.1016/0021-9681(87)90171-8

[17] M. M. Oken , R. H. Creech , D. C. Tormey , et al., “Toxicity and Response Criteria of the Eastern Cooperative Oncology Group,” American Journal of Clinical Oncology 5 (1982 Dec): 649–655. 7165009

[18] Centers for Disease Control and Prevention , “Assessing Your Weight,” (2022).

[19] T. Cederholm , G. L. Jensen , M. I. T. D. Correia , et al., “GLIM Criteria for the Diagnosis of Malnutrition – A Consensus Report From the Global Clinical Nutrition Community,” Journal of Cachexia, Sarcopenia and Muscle 10 (2019): 207–217. 30920778 10.1002/jcsm.12383 PMC6438340

[20] J. Arends , F. Strasser , S. Gonella , et al., “Cancer Cachexia in Adult Patients: ESMO Clinical Practice Guidelines☆,” ESMO Open 6 (2021 Jun): 100092. 34144781 10.1016/j.esmoop.2021.100092 PMC8233663

[21] R. D. Dolan , M. Alwahid , S. T. McSorley , et al., “A Comparison of the Prognostic Value of Composite Ratios and Cumulative Scores in Patients With Operable Rectal Cancer,” Scientific Reports 21, no. 10 (2020): 17965. 10.1038/s41598-020-73909-0 PMC7578034 33087753

[22] Q. Zhang , M. Song , X. Zhang , et al., “Association of Systemic Inflammation With Survival in Patients With Cancer Cachexia: Results From a Multicentre Cohort Study,” Journal of Cachexia, Sarcopenia and Muscle 12, no. 12 (2021): 1466–1476. 34337882 10.1002/jcsm.12761 PMC8718079

[23] S. van Buuren and K. Groothuis‐Oudshoorn , “mice: Multivariate Imputation by Chained Equations in R,” Journal of Statistical Software 45, no. 3 (2011): 1–67, 10.18637/jss.v045.i03.

[24] K. Fearon , F. Strasser , S. D. Anker , et al., “Definition and Classification of Cancer Cachexia: An International Consensus,” Lancet Oncology (Elsevier Ltd) 12 (2011): 489–495. 10.1016/S1470-2045(10)70218-7 21296615

[25] L. R. Brown , B. J. A. Laird , S. J. Wigmore , and R. J. E. Skipworth , “Understanding Cancer Cachexia and Its Implications in Upper Gastrointestinal Cancers,” Current Treatment Options in Oncology 23 (2022): 1732–1747, 10.1007/s11864-022-01028-1. 36269458 PMC9768000

[26] L. R. Brown , J. Sayers , M. S. Yule , et al., “The Prognostic Impact of pre‐Treatment Cachexia in Resectional Surgery for Oesophagogastric Cancer: A Meta‐Analysis and Meta‐Regression,” British Journal of Surgery 110 (2023): znad239–1711. 10.1093/bjs/znad239 PMC10638534 37527401

[27] C. M. Prado , J. R. Lieffers , L. J. McCargar , et al., “Prevalence and Clinical Implications of Sarcopenic Obesity in Patients With Solid Tumours of the Respiratory and Gastrointestinal Tracts: A Population‐Based Study,” Lancet Oncology 9 (2008): 629–635. 18539529 10.1016/S1470-2045(08)70153-0

[28] J. K. Kays , S. Shahda , M. Stanley , et al., “Three Cachexia Phenotypes and the Impact of Fat‐Only Loss on Survival in FOLFIRINOX Therapy for Pancreatic Cancer: Distinct Cachexia Phenotypes and Survival in PDAC,” Journal of Cachexia, Sarcopenia and Muscle 9 (2018): 673–684. 29978562 10.1002/jcsm.12307 PMC6104116

[29] J. Poisson , C. Martinez‐Tapia , D. Heitz , et al., “Prevalence and Prognostic Impact of Cachexia Among Older Patients With Cancer: A Nationwide Cross‐Sectional Survey (NutriAgeCancer),” Journal of Cachexia, Sarcopenia and Muscle 12 (2021): 1477–1488. 34519440 10.1002/jcsm.12776 PMC8718093

[30] K. R. Parashuram Rajapur and S. N. M.,. N. V. Dakshayani , “A Study of Sacralisatino of Fifth Lumbar Vertebra,” IJAR 5 (2017): 3718–3721.

[31] L. R. Brown , M. I. Ramage , R. D. Dolan , et al., “The Impact of Acute Systemic Inflammation Secondary to Oesophagectomy and Anastomotic Leak on Computed Tomography Body Composition Analyses,” Cancers 30, no. 15 (2023): 2577. 10.3390/cancers15092577 PMC10177546 37174044

[32] E. Cheng , J. Kirley , E. M. Cespedes Feliciano , and B. J. Caan , “Adiposity and Cancer Survival: A Systematic Review and Meta‐Analysis,” Cancer Causes & Control 33 (2022): 1219–1246. 35971021 10.1007/s10552-022-01613-7 PMC10101770

[33] B. J. A. Laird and R. J. E. Skipworth , “The Obesity Paradox in Cancer: Is Bigger Better?,” Journal of Cachexia, Sarcopenia and Muscle 13 (2022): 1440–1441. 35506563 10.1002/jcsm.13007 PMC9178354

[34] M. Kyrgiou , I. Kalliala , G. Markozannes , et al., “Adiposity and Cancer at Major Anatomical Sites: Umbrella Review of the Literature,” BMJ 28 (2017): j477. 10.1136/bmj.j477 PMC5421437 28246088

