# Supplementary material for: Comparison Between Single‐ and Multi‐slice Computed Tomography Body Composition Analysis in Patients With Oesophagogastric Cancer
Source: J Cachexia Sarcopenia Muscle. 2024 Dec 26;16(1):e13673. doi: 10.1002/jcsm.13673 (PMC11669944; doi:10.1002/jcsm.13673)
Supplement: Supplementary file 1 — Appendix S1: Body Composition Analysis. Appendix S2: Comparison of Clinicopathological Characteristics = Between Outliers and Non‐Outliers for Each Respective Tissue Type. Appendix S3: Bland–Altman Plots Showing Agreement Between Z‐Scores of Single‐ & Multi‐Slice Body Composition Estimates with Limits of Agreement (LoA) with BMI Colour Scale. Appendix S4: Comparison of Agreement Between Z‐Scores of Single‐ and Multi‐Slice Body Composition Estimates with Patients Stratified by BMI. Appendix S5: Comparison of Agreement Between Z‐Scores of Single‐ and Multi‐Slice Body Composition Estimate (Whole Cohort vs. Scaled Estimates). Appendix S6: Comparison of Association Between Single‐ and Multi‐Slice Body Composition and Overall Survival. Appendix S7: Supporting Information. [file JCSM-16-e13673-s001.docx]

**Appendix 1: Body Composition Analysis**

1. **Compared by Sex**

|  |  | Male  (n=342) | Female  (n=162) | *p* value |
| --- | --- | --- | --- | --- |
| *Multi-Slice Measures (T12-L4)* | | | | |
| Skeletal Muscle (cm^3^) | Mean (SD) | 2064 (543) | 1308 (365) | <0.001 |
| Muscle Radiodensity (HU) | Mean (SD) | 34 (11) | 35 (11) | 0.295 |
| Subcutaneous Fat (cm^3^) | Mean (SD) | 2408 (1461) | 2544 (1568) | 0.341 |
| Visceral Fat (cm^3^) | Mean (SD) | 2915 (1729) | 1369 (1068) | <0.001 |
| Intramuscular Fat (cm^3^) | Mean (SD) | 347 (161) | 276 (115) | <0.001 |
| *Single-Slice Measures (L3)* | | | | |
| Skeletal Muscle Area (cm^2^) | Mean (SD) | 136 (33) | 93 (21) | <0.001 |
| Muscle Radiodensity (HU) | Mean (SD) | 34 (11) | 34 (12) | 0.946 |
| Subcutaneous Fat (cm^2^) | Mean (SD) | 168 (98) | 189 (113) | 0.041 |
| Visceral Fat (cm^2^) | Mean (SD) | 200 (121) | 104 (79) | <0.001 |
| Intramuscular Fat (cm^2^) | Mean (SD) | 18 (9) | 17 (9) | 0.205 |

1. **Compared by Diagnosis of Cachexia**

|  |  | Cachexia  (n=221) | No Cachexia  (n=266) | *p* value |
| --- | --- | --- | --- | --- |
| *Multi-Slice Measures (T12-L4)* | | | | |
| Skeletal Muscle (cm^3^) | Mean (SD) | 1630 (520) | 1985 (622) | <0.001 |
| Muscle Radiodensity (HU) | Mean (SD) | 33 (12) | 35 (11) | 0.078 |
| Subcutaneous Fat (cm^3^) | Mean (SD) | 2222 (1505) | 2642 (1453) | 0.002 |
| Visceral Fat (cm^3^) | Mean (SD) | 2131 (1608) | 2693 (1767) | <0.001 |
| Intramuscular Fat (cm^3^) | Mean (SD) | 320 (156) | 327 (149) | 0.590 |
| *Single-Slice Measures (L3)* | | | | |
| Skeletal Muscle Area (cm^2^) | Mean (SD) | 111 (30) | 133 (36) | <0.001 |
| Muscle Radiodensity (HU) | Mean (SD) | 32 (11) | 35 (11) | 0.003 |
| Subcutaneous Fat (cm^2^) | Mean (SD) | 160 (107) | 187 (98) | 0.003 |
| Visceral Fat (cm^2^) | Mean (SD) | 152 (114) | 187 (120) | 0.001 |
| Intramuscular Fat (cm^2^) | Mean (SD) | 18 (9) | 17 (9) | 0.568 |

**Appendix 2: Comparison of Clinicopathological Characteristics = Between Outliers and Non-Outliers for Each Respective Tissue Type**

1. **Skeletal Muscle Based Body Composition Measurements**

|  |  | **Skeletal Muscle Area / Volume** | | | **Skeletal Muscle Radiodensity** | | |
| --- | --- | --- | --- | --- | --- | --- | --- |
|  |  | **Within LoA (n=486)** | **Outside LoA (n=18)** | ***p* value** | **Within LoA (n=488)** | **Outside LoA**  **(n=16)** | ***p value*** |
| **Age (years)** | **Median [IQR]** | 72.0 [64.0 to 80.0] | 70.5 [64.0 to 77.0] | 0.408 | 72.0 [63.8 to 80.0] | 69.0 [64.8 to 80.5] | 0.912 |
| **Sex** | **Male** | 328 (67.5) | 14 (77.8) | 0.448 | 332 (68.0) | 10 (62.5) | 0.599 |
|  | **Female** | 158 (32.5) | 4 (22.2) |  | 156 (32.0) | 6 (37.5) |  |
| **ASA** | **1** | 34 (7.0) | 1 (5.6) | 0.308 | 35 (7.2) | 0 (0.0) | 0.392 |
|  | **2** | 205 (42.2) | 6 (33.3) |  | 206 (42.2) | 5 (31.2) |  |
|  | **3** | 230 (47.3) | 9 (50.0) |  | 229 (46.9) | 10 (62.5) |  |
|  | **4** | 17 (3.5) | 2 (11.1) |  | 18 (3.7) | 1 (6.2) |  |
| **Height (m)** | **Median [IQR]** | 1.7 [1.6 to 1.8] | 1.7 [1.5 to 1.8] | 0.724 | 1.7 [1.6 to 1.8] | 1.7 [1.6 to 1.7] | 0.684 |
| **Weight (kg)** | **Median [IQR]** | 74.8 [62.0 to 88.0] | 84.7 [73.2 to 92.0] | 0.138 | 75.0 [62.3 to 88.0] | 67.7 [45.5 to 94.8] | 0.283 |
| **BMI (kg/m^2^)** | **Median [IQR]** | 26.0 [22.7 to 29.9] | 28.6 [25.5 to 36.1] | 0.060 | 26.2 [22.8 to 30.1] | 24.5 [17.8 to 28.6] | 0.202 |
| **Weight Loss** | **Median [IQR]** | 4.0 [0.0 to 7.0] | 5.0 [0.0 to 13.3] | 0.337 | 4.0 [0.0 to 8.0] | 4.0 [4.0 to 5.5] | 0.839 |
| **NLR** | **Median [IQR]** | 3.5 [2.3 to 5.8] | 3.9 [2.5 to 7.4] | 0.494 | 3.5 [2.3 to 5.6] | 5.4 [4.0 to 9.5] | 0.015 |
| **Cachexia** | **Yes** | 214 (45.5) | 7 (41.2) | 0.807 | 209 (44.3) | 12 (80.0) | 0.008 |
|  | **No** | 256 (54.5) | 10 (58.8) |  | 263 (55.7) | 3 (20.0) |  |
| **Stage** | **1** | 3 (0.7) | 0 (0.0) | 0.229 | 3 (0.7) | 0 (0.0) | 0.571 |
|  | **2** | 40 (8.8) | 3 (16.7) |  | 43 (9.4) | 0 (0.0) |  |
|  | **3** | 121 (26.6) | 7 (38.9) |  | 124 (27.1) | 4 (25.0) |  |
|  | **4** | 291 (64.0) | 8 (44.4) |  | 287 (62.8) | 12 (75.0) |  |

1. **Fat Based Body Composition Measurements**

|  |  | **SAT Area / Volume** | | | **VAT Area / Volume** | | | **IMAT Area / Volume** | | |
| --- | --- | --- | --- | --- | --- | --- | --- | --- | --- | --- |
|  |  | **Within LoA**  **(n=481)** | **Outside LoA (n=23)** | ***p value*** | **Within LoA (n=476)** | **Outside LoA (n=28)** | ***p value*** | **Within LoA**  **(n=481)** | **Outside LoA**  **(n=23)** | ***p value*** |
| **Age (years)** | **Median [IQR]** | 72.0 [64.0 to 80.0] | 69.0 [61.0 to 80.5] | 0.500 | 72.0 [63.0 to 80.0] | 73.5 [67.0 to 82.5] | 0.332 | 72.0 [63.0 to 80.0] | 74.0 [68.0 to 81.5] | 0.186 |
| **Sex** | **Male** | 331 (68.8) | 11 (47.8) | 0.041 | 324 (68.1) | 18 (64.3) | 0.681 | 326 (67.8) | 16 (69.6) | 1.000 |
|  | **Female** | 150 (31.2) | 12 (52.2) |  | 152 (31.9) | 10 (35.7) |  | 155 (32.2) | 7 (30.4) |  |
| **ASA** | **1** | 34 (7.1) | 1 (4.3) | 0.688 | 35 (7.4) | 0 (0.0) | 0.208 | 35 (7.3) | 0 (0.0) | 0.128 |
|  | **2** | 203 (42.2) | 8 (34.8) |  | 202 (42.4) | 9 (32.1) |  | 205 (42.6) | 6 (26.1) |  |
|  | **3** | 225 (46.8) | 14 (60.9) |  | 221 (46.4) | 18 (64.3) |  | 223 (46.4) | 16 (69.6) |  |
|  | **4** | 19 (4.0) | 0 (0.0) |  | 18 (3.8) | 1 (3.6) |  | 18 (3.7) | 1 (4.3) |  |
| **Height (m)** | **Median [IQR]** | 1.7  [1.6 to 1.8] | 1.7  [1.5 to 1.8] | 0.212 | 1.7  [1.6 to 1.8] | 1.6  [1.6 to 1.8] | 0.428 | 1.7  [1.6 to 1.8] | 1.7  [1.6 to 1.7] | 0.803 |
| **Weight (kg)** | **Median [IQR]** | 74.6 [62.0 to 87.3] | 89.9 [71.7 to 112.5] | 0.005 | 74.0 [61.6 to 87.4] | 86.0 [76.0 to 103.1) | <0.001 | 74.4 [61.5 to 87.6] | 86.9 [75.0 to 103.6] | <0.001 |
| **BMI (kg/m^2^)** | **Median [IQR]** | 25.9  [22.6 to 29.8] | 34.4  [26.4 to 37.2] | <0.001 | 25.9 [22.4 to 29.7] | 30.9 [27.9 to 36.2] | <0.001 | 25.9 [22.4 to 29.9] | 31.6 [26.8 to 36.4] | <0.001 |
| **Weight Loss** | **Median [IQR]** | 4.0  [0.0 to 7.0] | 6.0  [0.5 to 11.8] | 0.095 | 4.0  [0.0 to 7.0] | 6.0  [0.0 to 9.0] | 0.404 | 4.0  [0.0 to 7.0] | 3.8  [0.0 to 11.2] | 0.819 |
| **NLR** | **Median [IQR]** | 3.5  [2.3 to 5.8] | 3.6 [2.8 to 4.9] | 0.833 | 3.5 [2.3 to 5.8] | 3.6 [2.7 to 5.5] | 0.722 | 3.5 [2.3 to 5.8] | 3.9 [2.3 to 6.8] | 0.678 |
| **GLIM** | **Yes** | 209 (44.8) | 12 (57.1) | 0.371 | 206 (44.9) | 15 (53.6) | 0.436 | 212 (45.6) | 9 (40.9) | 0.827 |
| **Cachexia** | **No** | 257 (55.2) | 9 (42.9) |  | 253 (55.1) | 13 (46.4) |  | 253 (54.4) | 13 (59.1) |  |
| **Stage** | **1** | 3 (0.7) | 0 (0.0) | 0.528 | 3 (0.7) | 0 (0.0) | 0.459 | 3 (0.6) | 0 (0.0) | 0.867 |
|  | **2** | 40 (8.9) | 3 (13.6) |  | 39 (8.7) | 4 (16.0) |  | 42 (8.7) | 1 (4.3) |  |
|  | **3** | 124 (27.5) | 4 (18.2) |  | 123 (27.5) | 5 (20.0) |  | 122 (25.4) | 6 (26.1) |  |
|  | **4** | 284 (63.0) | 15 (68.2) |  | 283 (63.2) | 16 (64.0) |  | 283 (58.8) | 16 (69.6) |  |

**Appendix 3: Bland-Altman Plots Showing Agreement Between Z-Scores of Single- & Multi-Slice Body Composition Estimates with Limits of Agreement (LoA) with BMI Colour Scale.**

| **(A)** | 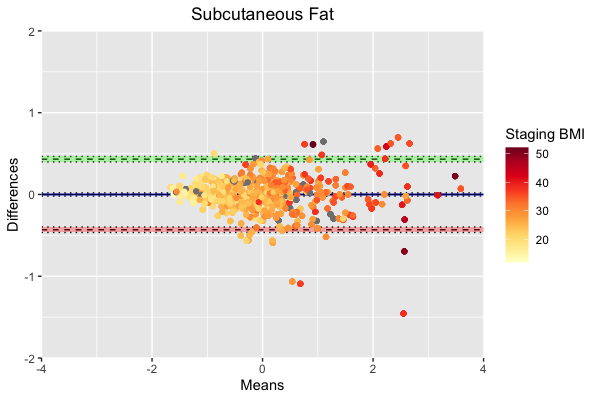 | |
| --- | --- | --- |
| **(B)** | 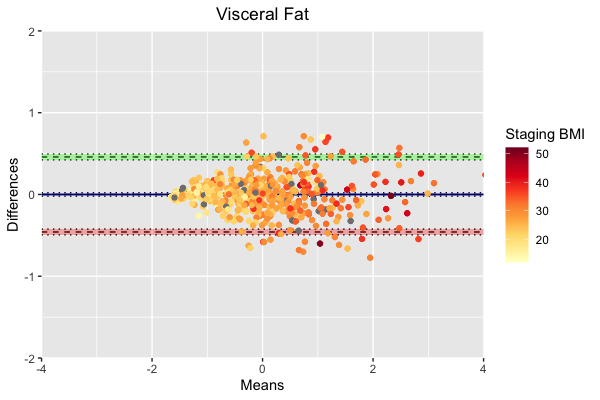 | |
| **(C)** | 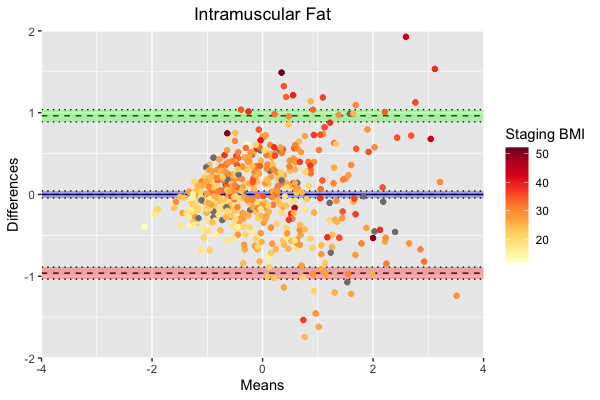 | |
|  | |  |

**Appendix 4: Comparison of Agreement Between Z-Scores of Single- and Multi-Slice Body Composition Estimates with Patients Stratified by BMI**

|  | Pearson Correlation | Bland Altman Analysis | | |
| --- | --- | --- | --- | --- |
|  |  | *Bias* | *Upper LoA* | *Lower LoA* |
| Skeletal Muscle  *(All Patients)* | 0.97  (*p*<0.001) | 0.00  (-0.02 to 0.02) | 0.48  (0.45 to 0.52) | -0.48  (-0.52 to -0.45) |
| *Obese Cohort (n=122)* | 0.97 (*p*<0.001) | -0.01 (-0.06 to 0.04) | 0.53 (0.45 to 0.62) | -0.55 (-0.64 to -0.47) |
| *Normal / Overweight Cohort (n=318)* | 0.96 (*p*<0.001) | -0.01 (-0.03 to 0.02) | 0.45 (0.40 to 0.49) | -0.46 (-0.51 to -0.42) |
| *Underweight Cohort (n=38)* | 0.94 (*p*<0.001) | 0.08 (0.00 to 0.16) | 0.53 (0.40 to 0.67) | -0.38 (-0.51 to -0.24) |
| Radiodensity *(All Patients)* | 0.93 (*p*<0.001) | 0.00 (-0.03 to 0.03) | 0.73  (0.68 to 0.79) | -0.73  (-0.79 to -0.68) |
| *Obese Cohort (n=122)* | 0.97 (*p*<0.001) | -0.12 (-0.17 to -0.07) | 0.41 (0.32 to 0.49) | -0.64 (-0.72 to -0.56) |
| *Normal / Overweight Cohort (n=318)* | 0.94 (*p*<0.001) | 0.01 (-0.03 to 0.04) | 0.65  (0.58 to 0.71) | -0.63  (-0.69 to -0.57) |
| *Underweight Cohort (n=38)* | 0.71 (*p*<0.001) | 0.33 (0.09 to 0.57) | 1.76 (1.35 to 2.18) | -1.11 (-1.53 to -0.70) |
| Subcutaneous Fat *(All Patients)* | 0.98  (*p*<0.001) | 0.00  (-0.02 to 0.02) | 0.43 (0.40 to 0.47) | -0.43 (-0.47 to -0.40) |
| *Obese Cohort (n=122)* | 0.96 (*p*<0.001) | 0.05 (0.00 to 0.10) | 0.64 (0.55 to 0.73) | -0.54 (-0.63 to -0.45) |
| *Normal / Overweight Cohort (n=318)* | 0.96 (*p*<0.001) | -0.03 (-0.05 to -0.01) | 0.35 (0.31 to 0.38) | -0.40 (-0.43 to -0.36) |
| *Underweight Cohort (n=38)* | 0.97 (*p*<0.001) | 0.03  (0.00 to 0.06) | 0.21 (0.16 to 0.26) | -0.15 (-0.20 to -0.10) |
| Visceral Fat *(All Patients)* | 0.97 (*p*<0.001) | 0.00 (-0.02 to 0.02) | 0.46 (0.42 to 0.49) | -0.46 (-0.49 to -0.42) |
| *Obese Cohort (n=122)* | 0.96 (*p*<0.001) | 0.01 (-0.05 to 0.06) | 0.60 (0.51 to 0.70) | -0.59 (-0.68 to -0.49) |
| *Normal / Overweight Cohort (n=318)* | 0.96 (*p*<0.001) | 0.00 (-0.03 to 0.02) | 0.42 (0.38 to 0.46) | -0.43 (-0.47 to 0.39) |
| *Underweight Cohort (n=38)* | 0.98 (*p*<0.001) | 0.02 (-0.03 to 0.06) | 0.30 (0.22 to 0.38) | -0.26 (-0.35 to -0.18) |
| Intramuscular Fat  *(All Patients)* | 0.86 (*p*<0.001) | 0.00 (-0.05 to 0.05) | 0.96 (0.89 to 1.04) | -0.96 (-1.04 to -0.89) |
| *Obese Cohort (n=122)* | 0.89 (*p*<0.001) | 0.29 (0.19 to 0.38) | 1.31 (1.15 to 1.47) | -0.74 (-0.90 to -0.58) |
| *Normal / Overweight Cohort (n=318)* | 0.88 (*p*<0.001) | -0.08 (-0.13 to -0.03) | 0.82 (0.73 to 0.90) | -0.97 (-1.06 to -0.89) |
| *Underweight Cohort (n=38)* | 0.90 (*p*<0.001) | -0.25 (-0.34 to -0.15) | 0.34 (0.17 to 0.51) | -0.83 (-1.00 to -0.66) |

**Appendix 5: Comparison of Agreement Between Z-Scores of Single- and Multi-Slice Body Composition Estimate (Whole Cohort vs. Scaled Estimates)**

|  | Pearson Correlation | Bland Altman Analysis | | |
| --- | --- | --- | --- | --- |
|  |  | *Bias* | *Upper LoA* | *Lower LoA* |
| Skeletal Muscle | 0.97  (*p*<0.001) | 0.00  (-0.02 to 0.02) | 0.48  (0.45 to 0.52) | -0.48  (-0.52 to -0.45) |
| *(Multi-Slice Scaled to Torso Length)* | 0.95 (*p*<0.001) | 0.00 (-0.03 to 0.03) | 0.62 (0.57 to 0.66) | -0.62 (-0.66 to -0.57) |
| *(Measurements Scaled to Height ^2^)* | 0.95 (*p*<0.001) | 0.00 (-0.03 to 0.03) | 0.64 (0.59 to 0.69) | -0.64 (-0.69 to -0.59) |
| Subcutaneous Fat | 0.98  (*p*<0.001) | 0.00  (-0.02 to 0.02) | 0.43 (0.40 to 0.47) | -0.43 (-0.47 to -0.40) |
| *(Multi-Slice Scaled to Torso Length)* | 0.97 (*p*<0.001) | 0.00 (-0.02 to 0.02) | 0.48 (0.45 to 0.52) | -0.48 (-0.52 to -0.45) |
| *(Measurements Scaled to Height ^2^)* | 0.97 (*p*<0.001) | 0.00 (-0.02 to 0.02) | 0.46 (0.42 to 0.49) | -0.46 (-0.49 to -0.42) |
| Visceral Fat | 0.97 (*p*<0.001) | 0.00 (-0.02 to 0.02) | 0.46 (0.42 to 0.49) | -0.46 (-0.49 to 0.42) |
| *(Multi-Slice Scaled to Torso Length)* | 0.98 (*p*<0.001) | 0.00 (-0.02 to 0.02) | 0.43 (0.40 to 0.46) | -0.43 (-0.46 to -0.40) |
| *(Measurements Scaled to Height ^2^)* | 0.97  (*p*<0.001) | 0.00  (-0.02 to 0.02) | 0.45  (0.41 to 0.48) | -0.45 (-0.48 to -0.41) |
| Intramuscular Fat | 0.88 (*p*<0.001) | 0.00 (-0.05 to 0.05) | 0.96 (0.89 to 1.04) | -0.96 (-1.04 to -0.89) |
| *(Multi-Slice Scaled to Torso Length)* | 0.87 (*p*<0.001) | 0.00 (-0.05 to 0.05) | 1.00 (0.92 to 1.08) | -1.00 (-1.08 to -0.92) |
| *(Measurements Scaled to Height ^2^)* | 0.86 (*p*<0.001) | 0.00  (-0.05 to 0.05) | 1.04 (0.96 to 1.12) | -1.04 (-1.12 to -0.96) |

Bland Altman analysis presented with 95% confidence intervals in parenthesis.

*Sensitivity Analyses – Scaling Multi-Slice Tissue Measurement by Torso Length*

Multi-slice measurements were scaled by torso length then sex-stratified z-score were re-derived and compared to z-scores from single-slice analysis. Correlations remained similarly strong to the un-scaled results (SKM: R=0.95 vs. 0.97, SAT: 0.97 vs. 0.97, VAT: 0.98 vs. 0.98, IMAT: 0.88 vs. 0.87). Following scaling, the limits of agreement for SKM were slightly broader (scaled LoA: ± 0.62 (95% CI: 0.57 - 0.66) vs. un-scaled LoA: ± 0.48 (95% CI: 0.45 - 0.52)). Differences between scaled and un-scaled analyses for VAT, SAT, IMAT measurements were minimal.

*Sensitivity Analyses – Normalising Tissue Measurements for Height*

Measurements from multi-slice and single-slice scans were then normalised for patient height squared where this data were available (n=484/504 , 96.0%). Correlations between multi-slice and single-slice measurements were comparable for all tissue types, but not improved following adjustment for height. The limits of agreement for SKM were again slightly broader, when compared to those of the unadjusted measurements (LoA: ± 0.64 (95% CI: 0.59 - 0.69) vs. un-scaled LoA: ± 0.48 (95% CI: 0.45 - 0.52)). Minimal differences were evident between scaled and un-scaled analyses for VAT, SAT, IMAT measurements.

**Appendix 6: Comparison of Association Between Single- and Multi-Slice Body Composition and Overall Survival**

1. **Curative Subgroup**

|  | **L3 Single-Slice (Cross-Sectional Area)** | | | | **Multi-Slice (Volume)** | | | |
| --- | --- | --- | --- | --- | --- | --- | --- | --- |
|  | **Univariable HR (95% CI)** | ***p* value** | **Multivariable HR (95% CI)** | ***p* value** | **Univariable HR (95% CI)** | ***p* value** | **Multivariable HR (95% CI)** | ***p* value** |
| **SKM  Area / Volume** | 0.82  (0.63-1.06) | 0.133 | 0.65 (0.43-0.97) | 0.038 | 0.84 (0.65-1.08) | 0.169 | 0.67 (0.45-0.99) | 0.045 |
| **SKM**  **Radio-density** | 1.00 (0.73-1.38) | 0.977 | 0.97 (0.70-1.34) | 0.852 | 0.97 (0.70-1.34) | 0.834 | 0.95 (0.68-1.32) | 0.755 |
| **SAT**  **Area / Volume** | 1.03 (0.74-1.44) | 0.851 | 1.04 (0.76-1.44) | 0.790 | 1.05 (0.77-1.45) | 0.742 | 1.05 (0.77-1.43) | 0.757 |
| **VAT**  **Area / Volume** | 0.93 (0.71-1.23) | 0.633 | 0.94 (0.71-1.24) | 0.673 | 0.94 (0.71-1.24) | 0.639 | 0.93 (0.71-1.23) | 0.619 |
| **IMAT**  **Area / Volume** | 0.97 (0.74-1.28) | 0.854 | 0.97 (0.73-1.28) | 0.832 | 0.97 (0.74-1.27) | 0.826 | 0.97 (0.74-1.27) | 0.833 |

Each tissue measurement has modelled in isolation with adjustment for confounders but not for other tissue measurements. HR: Hazard Ratio. CI: Confidence Interval. SKM: Skeletal Muscle. SAT: Subcutaneous Adipose Tissue. VAT: Visceral Adipose Tissue. IMAT: Intramuscular Adipose Tissue. L3: 3^rd^ Lumbar Vertebra

1. **Non-Curative Subgroup**

|  | **L3 Single-Slice (Cross-Sectional Area)** | | | | **Multi-Slice (Volume)** | | | |
| --- | --- | --- | --- | --- | --- | --- | --- | --- |
|  | **Univariable HR (95% CI)** | ***p* value** | **Multivariable HR (95% CI)** | ***p* value** | **Univariable HR (95% CI)** | ***p* value** | **Multivariable HR (95% CI)** | ***p* value** |
| **SKM  Area / Volume** | 0.78 (0.68-0.88) | <0.001 | 0.67 (0.58-0.79) | <0.001 | 0.82 (0.73-0.94) | 0.003 | 0.71 (0.60-0.83) | <0.001 |
| **SKM**  **Radio-density** | 0.95 (0.85-1.06) | 0.338 | 0.97 (0.87-1.09) | 0.610 | 0.98 (0.88-1.10) | 0.753 | 1.00 (0.89-1.12) | 0.974 |
| **SAT**  **Area / Volume** | 0.83 (0.74-0.93) | 0.002 | 0.83 (0.74-0.93) | 0.001 | 0.84  (0.75-0.95) | 0.004 | 0.84 (0.75-0.94) | 0.003 |
| **VAT**  **Area / Volume** | 0.83 (0.74-0.93) | 0.002 | 0.83 (0.74-0.93) | 0.002 | 0.82 (0.73-0.93) | 0.001 | 0.82 (0.73-0.93) | 0.001 |
| **IMAT**  **Area / Volume** | 0.97 (0.86-1.08) | 0.560 | 0.95  (0.85-1.07) | 0.391 | 0.98 (0.87-1.10) | 0.709 | 0.96  (0.86-1.07) | 0.466 |

Each tissue measurement has modelled in isolation with adjustment for confounders but not for other tissue measurements. HR: Hazard Ratio. CI: Confidence Interval. SKM: Skeletal Muscle. SAT: Subcutaneous Adipose Tissue. VAT: Visceral Adipose Tissue. IMAT: Intramuscular Adipose Tissue. L3: 3^rd^ Lumbar Vertebra

**Appendix 7:**

1. **Non-Obese Subgroup**

|  | **L3 Single-Slice (Cross-Sectional Area)** | | | | **Multi-Slice (Volume)** | | | |
| --- | --- | --- | --- | --- | --- | --- | --- | --- |
|  | **Univariable HR (95% CI)** | ***p* value** | **Multivariable HR (95% CI)** | ***p* value** | **Univariable HR (95% CI)** | ***p* value** | **Multivariable HR (95% CI)** | ***p* value** |
| **SKM  Area / Volume** | 0.57  (0.47 - 0.69) | <0.001 | 0.58 (0.48 - 0.71) | <0.001 | 0.62  (0.50 - 0.76) | <0.001 | 0.61 (0.50 - 0.76) | <0.001 |
| **SKM**  **Radio-density** | 0.93  (0.80 - 1.07) | 0.284 | 0.95 (0.82 - 1.10) | 0.492 | 0.97 (0.83 - 1.13) | 0.698 | 0.99 (0.85-1.15) | 0.867 |
| **SAT**  **Area / Volume** | 0.68 (0.56 - 0.81) | <0.001 | 0.68 (0.57 - 0.81) | <0.001 | 0.70 (0.58 - 0.84) | <0.001 | 0.70 (0.58 - 0.85) | <0.001 |
| **VAT**  **Area / Volume** | 0.83 (0.70 - 0.99) | 0.040 | 0.82 (0.69 - 0.98) | 0.030 | 0.79 (0.66 - 0.95) | 0.014 | 0.77 (0.64 - 0.93) | 0.008 |
| **IMAT**  **Area / Volume** | 0.88 (0.77 - 1.01) | 0.061 | 0.86 (0.75 - 0.99) | 0.042 | 0.86  (0.74 - 1.01) | 0.060 | 0.84 (0.72 - 0.98) | 0.029 |

Each tissue measurement has modelled in isolation with adjustment for confounders but not for other tissue measurements. HR: Hazard Ratio. CI: Confidence Interval. SKM: Skeletal Muscle. SAT: Subcutaneous Adipose Tissue. VAT: Visceral Adipose Tissue. IMAT: Intramuscular Adipose Tissue. L3: 3^rd^ Lumbar Vertebra

1. **Obese Subgroup**

|  | **L3 Single-Slice (Cross Sectional Area)** | | | | **Multi-Slice (Volume)** | | | |
| --- | --- | --- | --- | --- | --- | --- | --- | --- |
|  | **Univariable HR (95% CI)** | ***p* value** | **Multivariable HR (95% CI)** | ***p* value** | **Univariable HR (95% CI)** | ***p* value** | **Multivariable HR (95% CI)** | ***p* value** |
| **SKM  Area / Volume** | 0.69 (0.48 - 0.99) | 0.045 | 0.66 (0.46 - 0.94) | 0.022 | 0.80 (0.57 - 1.12) | 0.199 | 0.72 (0.52 - 1.00) | 0.048 |
| **SKM**  **Radio-density** | 0.93  (0.74 - 1.15) | 0.490 | 0.90 (0.72 - 1.12) | 0.333 | 0.90 (0.72 - 1.12) | 0.331 | 0.87 (0.70 - 1.09) | 0.230 |
| **SAT**  **Area / Volume** | 1.19 (0.97 - 1.45) | 0.100 | 1.24 (1.01 - 1.52) | 0.043 | 1.24 (1.02 - 1.51) | 0.032 | 1.29 (1.05 - 1.57) | 0.015 |
| **VAT**  **Area / Volume** | 0.88 (0.68 - 1.14) | 0.327 | 0.89 (0.68 - 1.17) | 0.404 | 0.92 (0.71 - 1.19) | 0.524 | 0.90 (0.69 - 1.19) | 0.467 |
| **IMAT**  **Area / Volume** | 1.08 (0.88 - 1.33) | 0.436 | 1.06 (0.86 - 1.32) | 0.577 | 1.16 (0.96 - 1.40) | 0.130 | 1.14 (0.93 - 1.39) | 0.192 |

Each tissue measurement has modelled in isolation with adjustment for confounders but not for other tissue measurements. HR: Hazard Ratio. CI: Confidence Interval. SKM: Skeletal Muscle. SAT: Subcutaneous Adipose Tissue. VAT: Visceral Adipose Tissue. IMAT: Intramuscular Adipose Tissue. L3: 3^rd^ Lumbar Vertebra
